# Strategies to facilitate implementation and sustainability of large system transformations: a case study of a national program for improving quality of care for elderly people

**DOI:** 10.1186/1472-6963-14-401

**Published:** 2014-09-18

**Authors:** Monica Elisabeth Nyström, Helena Strehlenert, Johan Hansson, Henna Hasson

**Affiliations:** Department of Learning, Informatics, Management and Ethics, Medical Management Centre, Karolinska Institutet, SE 171 77 Stockholm, Sweden; Department of Public health and Clinical medicine, Epidemiology and Global health, Umeå University, SE 901 87 Umeå, Sweden; Centre for Epidemiology and community medicine, Stockholm county council, SE 171 29 Stockholm, Sweden

## Abstract

**Background:**

Large-scale change initiatives stimulating change in several organizational systems in the health and social care sector are challenging both to lead and evaluate. There is a lack of systematic research that can enrich our understanding of strategies to facilitate large system transformations in this sector. The purpose of this study was to examine the characteristics of core activities and strategies to facilitate implementation and change of a national program aimed at improving life for the most ill elderly people in Sweden. The program outcomes were also addressed to assess the impact of these strategies.

**Methods:**

A longitudinal case study design with multiple data collection methods was applied. Archival data (n = 795), interviews with key stakeholders (n = 11) and non-participant observations (n = 23) were analysed using content analysis. Outcome data was obtained from national quality registries.

**Results:**

This study presents an approach for implementing a large national change program that is characterized by initial flexibility and dynamism regarding content and facilitation strategies and a growing complexity over time requiring more structure and coordination. The description of activities and strategies show that the program management team engaged a variety of stakeholders and actor groups and accordingly used a palate of different strategies. The main strategies used to influence change in the target organisations were to use regional improvement coaches, regional strategic management teams, national quality registries, financial incentives and annually revised agreements. Interactive learning sessions, intense communication, monitor and measurements, and active involvement of different experts and stakeholders, including elderly people, complemented these strategies. Program outcomes showed steady progress in most of the five target areas, less so for the target of achieving coordinated care.

**Conclusions:**

There is no blue-print on how to approach the challenging task of leading large scale change programs in complex contexts, but our conclusion is that more attention has to be given to the multidimensional strategies that program management need to consider. This multidimensionality comprises different strategies depending on types of actors, system levels, contextual factors, program progress over time, program content, types of learning and change processes, and the conditions for sustainability.

## Background

World-wide, national authorities have tried to implement initiatives to improve quality and efficiency in their health and social care systems, with various degree of success e.g. [1–3]. As in several other countries, Swedish national authorities have tried to influence healthcare providers by delivering information on recommended practices, policy documents, descriptions of visions and areas in need of improvement, the latter sometimes accompanied with indicators for monitoring and evaluation. However, as means of improving health and social care the use of a passive diffusion of information approach has not produced the desired changes [4]. There is a need for more active implementation strategies [5–7]. Ferlie and Shortell [8] argued that unless policy and decision makers consider and implement a coherent and multilevel approach to change there is a low chance to improve the quality of a nation’s healthcare system. As such, no single change strategy fits all situations and a mixture of strategies are often called for when changing health care practice [9].

Many studies of changes in health and social care deal with relatively small-scale change initiatives involving a few organizations or single strategies e.g. [10, 11]. Large-scale change initiatives seeking to stimulate change in several organizational systems are more challenging to evaluate. Prior studies in this field have often lacked systematic analyses [12, 13]. Large-system transformations (LST) is defined as: “interventions aimed at coordinated, system wide change affecting multiple organizations and care providers, with the goal of significant improvement in the efficiency of health care delivery, the quality of patient care, and population-level patient outcomes.” [12], p. 422. LST will be used in this article to refer to large-scale change programs and quality improvement attempts. Gaining more insight into different approaches to LST, its features, strategies and outcomes can enrich our understanding of strategies to facilitate the improvement and change of large and complex health care systems.

### Factors influencing the success of LSTs in healthcare

**Figure 1:**
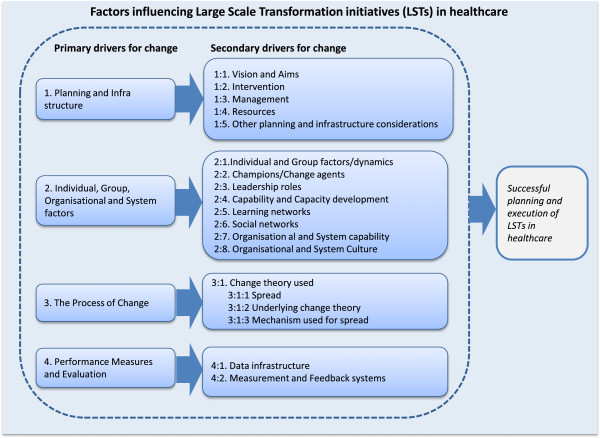
**A model over factors influencing Large Scale Transformation initiatives in healthcare.** Adapted from the model over large scale improvement initiatives by Perla et al. [15].

Previous empirical studies dealing with implementation of LST discuss success factors e.g. [14], change driving mechanisms e.g. [15], and/or contextual conditions e.g. [16] important for LST programs, but few frameworks are available to describe strategies for enhancing LSTs. A recent literature review of LSTs in healthcare introduces a model with four primary and 15 secondary drivers for change [15] that can be used to aid program planning and implementation. The model, presented in Figure 1, provides a framework for understanding of factors influencing the implementation of LSTs.

The driver ‘Planning and infrastructure’ includes vision and aim; carefully developed interventions; solid management; and sufficient resources, both in central administration and in participating organizations. This driver focuses on program organization and the clarity and content of the program. The associated secondary drivers mirror many normative advices from the change and project management literature. Studies have acknowledged the importance to ensure a solid organizational foundation and to assure that appropriate organizational and contextual conditions are in place or are being developed when launching LSTs in healthcare e.g. [17].

The driver ‘*Considering individual, group, organizational, and system factors’* include eight secondary drivers: individual and group factors and their dynamics; champions and change agents; leadership roles; capability and capacity development; learning networks; social networks; organizational and system capability; and organizational and system culture. This primary driver reflects the embedded complexity of the health or social care system and emphasizes the need for good system knowledge and actor strategies at multiple system levels. The review also highlights the involvement of several actors and consideration of system factors, for example a study identifying success factors of the Kaiser Permanente’s Performance Improvement System [14, 18]. Results consistent with the first two primary drivers can for example be found in a study of a women's health program in Michigan, where widely accepted standards and demonstrated value of the innovations, committed leaders/champions, and a participatory culture were important factors for success [19].

The driver ‘*Process of change’* has a secondary driver, the chosen change theory, which in turn has three dimensions: spread; underlying change theory; and the mechanisms used for spread. The model is vague on this driver, leaving room for interpretation on how to address the underlying change theory. Finally, driver four, ‘*Performance measures and evaluation’*, contains two secondary drivers: data infrastructure and measurement and feedback systems. This driver is connected to monitoring and evaluation of the various parts of an LST program and to the enhancement and use of feedback.

Other studies provide examples of all four drivers in the model. Lucas and colleagues [20] identified five interactive elements critical to LSTs in the US health care systems: impetus to transform; leadership commitment to quality; improvement initiatives that actively engage staff in meaningful problem solving; alignment to achieve consistency of organization goals with resource allocation and actions at all levels of the organization; and integration to bridge traditional intra-organizational boundaries among individual components. These elements were suggested to drive change by affecting the health care organisations’ mission, vision, strategies, culture, infrastructure, and operational functions and processes. A review of models for successful implementations [21] underlined the need for systematic approaches with focus on achieving a solid preparation and planning that involves ensuring readiness to change, building implementation structures and planning for interventions, support, feedback and evaluation – but also learning from experiences. However, many change programs start with less rigorous and systematic preparation and build structures and strategies as the process proceeds over time, a mixture of emergent and deliberate strategies where performance consensus becomes important [22]. This mixture of intended and emergent strategies is probably more common for LSTs since both interventions and target context are multidimensional, dynamic and complex.

### Leading and managing LSTs

Studies on factors influencing LSTs provide general frameworks for change agents (e.g. program management and project teams) to consider when planning for LST programs. Less information is available to show how to actually lead LSTs in complex contexts and how to choose and execute strategies to facilitate implementation and change processes. The perspective of the change agents has often been overlooked in research on development in healthcare [23]. Therefore, research from other areas, like change management, can provide more detailed input. For instance, basic assumptions on change and how such assumptions influence the core change strategy or strategies used by change agents have been described by de Caluwé and Vermaak [24, 25]. They illustrate five basic paradigms of change approaches: the blue-print approach (rational planning); the yellow-print approach (stakeholder involvement and negotiation); the green-print approach (interactive learning); the red-print approach (focus on individual’s needs and motivation); and the white-print approach (emergent and less controllable change). A prior study of a large-scale health promoting program showed that the program management’s use of these basic change approaches during implementation was not a one-choice option but rather the use of multiple strategies that varied over program phases [26]. Besides the focus on preventive actions, the program management team also faced challenges such as defining intermediate and end goals and clarifying the roles of each member of the team [27]. Different teams of change agents may also chose different approaches to enhance LSTs in health care depending on the context (e.g. organization, condition, patient group, staff) and may thereby vary in their success rates [28]. Strategies for overcoming resistance to change have been proposed, where change agents can choose to, for example, provide education; facilitate and support; and/or negotiate and come to an agreement [29, 30].

The few prior studies found on change agents’ strategies to facilitate the implementation and change process involved in LSTs provided a rationale for the current study. Accordingly, the purpose of the study was to examine the characteristics of core activities and strategies to facilitate implementation and change applied by the program management of a national program aimed at improving life for the most ill elderly people in Sweden. The program outcomes were also addressed to assess the impact of these strategies.

## Methods

A multi-method, longitudinal research design has been suggested as appropriate for studies of large-scale programs [2]. Richly described case studies addressing embedded change mechanisms in different contexts are advised [31]. Accordingly, a case study research design with multiple data collection methods was applied.

### Case characteristics

A national program for improving quality of care for elderly people in Sweden, the “Better life for the most ill elderly people program” (Be-Life program, 2010–2014) form the basis for the study. The Be-Life program addresses hospital care, primary care and eldercare organized by both public, private not-for profit and private for-profit organizations. As such, the program represents an LST attempt that can shed light on the strategic choices made to facilitate the program’s implementation in a complex setting with a large variation in its sub-systems.

The Swedish government is responsible for overall health and medical care policy, while the responsibilities for the provision of health care is decentralized to local and regional authorities. There are 21 regional authorities (county councils) that are responsible for providing primary care and specialized healthcare and 290 local authorities (municipalities) responsible for delivering social care and home health care. All these self-governing authorities are involved in the Be-life program.

The program aimed to improve the quality of care for older adults with complex health issues by improving cooperation between municipalities and county councils and streamline the use of resources in line with patient needs. There were also attempts to achieve a holistic frame of reference consistent with other national agreements by incorporating the aims to increase the use of national quality registry data for improvement in health and social care, and to build sustainable regional support structures for and competence in quality improvement.

The previous model used by the government for distributing improvement grants in which municipalities and county councils applied for funding had failed to produce intended outcomes. Therefore, the government had decided to make a successive shift towards incentive grants linked to performance-based targets. This induced an agreement between the Ministry of Health and Social Affairs and the Swedish Association of Local Authorities and Regions (SALAR), the latter an association representing the governmental, professional, and employer related interests of municipalities, county councils and regions. SALAR was given responsibility and funding to coordinate and support the regions in working towards the program goals.

During the first two years, the financial incentives part of the agreement focused on utilizing national quality registries for preventive and palliative care. The registries contain physician-level data regarding diagnoses, treatments, and outcomes, with the aim to be used for decision making, research and quality improvement e.g. [32]. Two registries, the Palliative Registry Sweden and Senior Alert, were initially included in the program. The Palliative Registry (initiated in 2006) uses a questionnaire to register how the deceased person’s needs for care were fulfilled during the last period of life, regarding for example pain reliefs. The preventive focused Senior Alert registry (initiated in 2008) centres on falling accidents, nutrition, decubitus ulcers, and oral health. The aim is to have risk assessments performed on a regular basis for each elderly person under care. Senior Alert uses several types of indexes (e.g. Downtown Fall Risk Index, MNA Mini Nutritional Assessment).

A study of the needs of the most ill elderly people commissioned by SALAR resulted in three additional performance-based target areas in the 2012 agreement and onwards: dementia care, pharmacological treatment and coordinated health and social care. For dementia care, performance bonuses were based on the use of two additional national quality registries, the registry for Behavioural and Mental Symptoms in Dementia (BPSD) and the Swedish Dementia Registry (SveDem). The SveDem registry (initiated in 2007) focuses on dementia and uses the instrument Qualid - Quality of life during severe dementia. Additional indicators for old people at special housing units are also registered (e.g. activity level, medicines, person-centred care, measures taken for protection and re-straining activities, such as alarms). The BPSD registry (initiated in 2010) registers information on psychological and behavioural symptoms for people with dementia (e.g. hallucinations, sleep disruptions, depression) and supports analyses and activity plans made for improving a patient’s situation.

In the new target area of pharmacological treatment, financial incentives were given for reducing inappropriate medications and/or combinations of medications (replaced by anti-inflammatory medications in the 2013 agreement) and inappropriate medications used to treat psychoses. Two indicators were used to measure coordination of health and social care: ‘avoidable inpatient care’ and ‘readmission within 30 days’. The indicator ‘avoidable in-patient care’ encompasses “unnecessary” admissions to hospital care due to a number of specified diagnoses assumed to be preventable by sufficient non-institutional care. Among the included diagnoses are asthma, diabetes, anaemia, high blood pressure, cardiac failure, and chronic obstructive pulmonary disease. The indicator ‘readmission within 30 days’ aims at both planned and acute hospital care (regardless of diagnosis), and is assumed to indicate cases when patients are discharged from hospitals too soon (with regard to their health status), inadequate pharmacological treatment, lack of information to patients, or insufficient care and follow-ups after discharge. Improvements within other target areas were assumed to have long-term effect on both these indicators, but direct measures were also needed to reduce avoidable hospital admissions and readmissions.

Designing valid indicators for pharmacological treatment and coordinated care was a challenging task. The basic idea was that indicators in these areas should capture how well municipalities and county councils managed cooperation and coordination of care, keeping the individual patient’s situation in focus. To facilitate regional improvement work the agreements comprised funding for three to six improvement coaches per region. A special initiative geared toward higher-level managers and key actors in municipalities and county councils called “From word to action – the practice of leadership” (Practice Leadership) was included in the 2012 agreement.

From 2012 and onwards, the agreement stated basic requirements that municipalities and county councils had to meet to qualify for financial incentives. Requirements included political resolution on regional action plans for improvements in care services for older adults with complex health conditions and documentation of a management system for systematic quality work in accordance with the National Board of Health and Welfare’s regulations. The estimated costs for the program was in 2010 294 MSEK (32.3 M€ calculated 2013-12-01); in 2011 296 MSEK (32.6 M€) and in 2012 1261 MSEK (138.4 M€). More detailed information on the program can be found on the Ministry of Health and Social Affair’s and SALAR’s websites (http://www.regeringen.se/sb/d/14622; http://www.skl.se/4.5e95253d14642b207ee4a7b.html).

### Data collection

The study covers the period from 2008 to 2013. Data was collected between October 2011 and December 2013 and consists of interviews, observations, documents, and data from national quality registries. Results on the preparation period (2008–2009) and the first two years of program implementation (2010–2011) were based on retrospective data (documentation and interviews).

Semi-structured interviews with key stakeholders at the national level were conducted by the first and the second author. The informants represented the Ministry of Health and Social Affairs (n = 2), the program management team (n = 3), and strategic functions at SALAR (n = 6). The criteria for selecting informants were involvement in the program from its on-set. The interviews lasted between 45 and 75 minutes. They were recorded and transcribed verbatim. The questions addressed six themes: program background, interventions and activities, strategies, conditions for learning and change, reactions and results, and future plans for the program. Participation was voluntary and based on informed consent.

The project documentation was gathered from the Ministry of Health and Social Affairs, the National Board of Health and Welfare, SALAR, and the Be-Life program’s internal and external websites. The documentation included newsletters, the project plan, annual action plans and result reports, monthly internal reports from the program management, agendas and notes from seminars, workshops and meetings, including Power-point presentations, all together 70 documents. E-mail communications at the program’s internal website were also part of the data set (700 forum posts 2010–2013). Other relevant documents (25 documents; e.g. agreements between the Ministry of Health and Social Affairs, SALAR; government assignments; the Swedish Agency for Public Management’s annual program evaluation reports; reports from the National Board of Health and Welfare), were collected from the respective web sites. In total, 795 documents were gathered.

During 2012 and 2013 non-participant observations of key project activities were made on twenty-three occasions by one or two researchers. These sessions included seven workshops for regional improvement coaches and five national seminars aimed at regional leaders in health and social care held by the project team (21 full days in total). Three full-day dissemination conferences and eight telephone conferences were also observed. During the observations the activities and reactions from participants was noted using a common format consisting of notes on time, type of event, type of activities, description of activities, actors involved, and comments.

The program’s outcomes were collected at municipality and county council level from the national quality registries for relevant indicators. The indicators were related to the target levels for performance bonuses defined in the agreement. The data was obtained in December 2013 and covered the period of 2009–2013.

### Data analysis

The interviews, observation protocols and documents were analysed using qualitative content analysis [33–35]. The analysis identified parts of the text that described program activities and important events that subsequently were categorized according to type, intention or goal, involved actors, and finally chronologically ordered. The program management informants also described the strategies they used and these were treated in a separate category. The information was compiled in a detailed chronological matrix of activities, intentions and actors, which constituted the base for the identification of strategies. At this level of analysis we defined a strategy as a cluster of similar activities related to an intention or aiming for a goal. We then identified the intentions and intermediate goals (i.e. goals that if achieved would aid the progress towards the programs main goals) and types of action strategies connected to those in an iterative process comparing goals and strategies over data sources. This procedure generated ten proposed strategies that were validated by the program management team during an interactive session. The definitions of two strategies were slightly re-formulated to fit the comments of the team. Descriptive statistics were used to analyse the quantitative data.

The project was partly financed by government money dedicated for the eldercare area and distributed via SALAR with no restrictions impending the research content or publications. The Regional Ethics Committee in Stockholm has granted ethical approval for the study [ref no. 2011/5:11].

## Results

### Core activities of the Be-Life program

The identified core activities of the program are organized into three program phases: preparation; initiation and early implementation; and implementation.

#### Preparation phase 2008–2009

The main focus during this phase was to explore and obtain stakeholder motivation, engagement and involvement, and to negotiate, decide and initiate the program. The main challenges, as described by the program management, was to get politicians’ and higher decision makers’ acceptance and understanding of the potential role of national quality registries and regional improvement coaches in improving quality of care for the most ill elderly people.

The discussions between the Ministry of Health and Social Affairs and SALAR about an agreement to introduce performance based financial incentives were initiated in 2008. The increased transparency of the national comparisons (i.e. publishing of results of quality measurements from all health and social care providers) introduced in 2006, together with new national quality registries targeting care for elderly people had provided insights into the performance of regions, municipalities and private organizations.

SALAR suggested that quality registries should be included in the Be-life program agreement and that regional improvement coaches should be used to support the use of registries. A program director with previous experience in quality improvement and coordination of care was recruited in 2009 to form and negotiate the up-coming agreement (signed by both parties in February 2010).

#### Initiation and early implementation phase 2010–2011

The program’s main content, goals and involved actors were negotiated and decided on during this phase. Several fora for communication were initiated. The program structure and approaches to implementation and facilitation were gradually formed, based on assumptions on the importance of interactive learning and a high degree of transparency. Program actions were characterized by a creative trial-error approach. As the management team gradually formed and more actors became involved, vague program strategies were specified and central problem areas identified (e.g. defining measurement strategies). The regional support structure consisting of improvement coaches was put into action. The number and frequency of program activities increased during this phase and implementation in the regions accelerated. Central agreements were annually revised and the first two agreements shared a similar content and scope.

##### Collaboration with national quality registries

Collaboration between the program management team and the Senior Alert and Palliative Registry Sweden were established in regular strategic meetings. Additional meetings with the two dementia registries that were to join the program in 2012 were held, as well as joint meetings with all four registries. A main focus during this phase was providing IT-solutions for easy and open access to the registries’ outcome data.

##### Support to regions and regional actors

To provide support for the regional improvement coaches in their assignment to locally implement the utilization of quality registries SALAR initiated a national network platform for the improvement coaches. An important aim of the network was to enable sharing of experiences. Eight network meetings were held during 2010 and 2011. In addition, a web-based, interactive project site was launched to facilitate experience-sharing and contacts between meetings.

##### Information and communication

An important aspect of SALAR’s work was to communicate the central program messages to all involved target groups. The program management team presented the program at national conferences, seminars and exhibitions, arranged regional conferences, seminars and educational activities and made numerous visits to regions and took part in many regional and local activities. A senior communication advisor was hired and a communication strategy formulated in 2011. SALAR’s established network of regional health and social care directors was another node used for the launch of and communication about the program.

##### Monitoring the external and internal environment

Another important feature during this phase was to monitor the external environment and gain relevant knowledge (and competence) for the program management team. Team members arranged conferences and international study visits to learn about large system change and patient involvement. The program director was a member of the government’s national coordinator team for the care of elderly people. There were ongoing discussions and coordination with the National Board of Health and Welfare and with other governmental assignments in the eldercare area. Efforts were made to monitor the internal SALAR environment to track other relevant programs and projects in order to optimize coordination.

##### Building a management team

The program management team expanded and new members were employed. In spring 2010, a sub-project leader was hired to manage SALAR’s support to the regional improvement coaches. In 2011 an administrator, a communicator, and two more sub-project leaders were recruited.

##### Internal program support, monitoring, evaluation and feedback

Mentoring support for the program management team played an important role from the start of the program. Long-term support consisted of critical friends (experts at SALAR, international experts on large-system change and improvement). In addition, individuals with specific competences were engaged on short term basis when needed. A reference group of elderly citizens was established. The government commissioned the Swedish Agency for Public Management to perform yearly follow ups and evaluations. Researchers in several universities were asked to follow the program and relate various aspects to scientific areas of interest.

#### Implementation phase 2012–2013

In 2012, the implementation process intensified. The agreements for 2012 and 2013 involved approximately four times more funding per year as the previous ones and included three new target areas: dementia, pharmacological treatment and coordinated care. The number and scope of program activities increased, more people and target groups became involved and the external communication multiplied. At the same time, the needs for coordination of program activities increased, and at the end of 2013 more discussions on how to sustain the program were detected.

##### Collaboration with national quality registries

Two dementia care registries were included in the program. During this period, the program management team continued to regularly invite the registries to strategic meetings. They also worked with the registries to coordinate and develop a web portal for easy access to outcome data.

##### Support to regions and regional actors

The 2012 agreement involved a shift in the improvement coach role, from supporting the mere use of registries towards facilitating improvement efforts based on registries and indicators. This shift influenced the activities within the improvement coaches’ network. Six network meetings were held during the period, along with coaching support via telephone or video conferences when needed. The sub-project ‘Practice Leadership’ launched in February 2012 was designed to inspire, engage and support the regional management levels when planning for and leading the regional work. Motivation was enhanced by the use of real world examples and a focus on the individual elderly person and his/her situation. International experts on large-scale change and improvement were invited to meetings with regional actors. As a part of the Practice Leadership initiative, a national support structure of established county council development centres was initiated with the aim to provide strategic support to the regional strategic management teams.

##### Information and communication

The 2012 agreement was launched at a national conference in January 2012, jointly arranged by SALAR and the Ministry of Health and Social Affairs. The intention was to send a clear message to all regions that the program had a strong backing at national level. Representatives from the program management team and the ministry visited municipalities and county councils together, and took part in various regional and local activities. Three conferences that focused especially on managers and medical advisors in primary care were arranged, addressing the challenge to engage these (and other) groups in primary care. Methods for program communication were further developed during this phase, for example the production of short videos and the use of social media such as Twitter and blogs. Special emphasis was put on presenting good local examples of improvement efforts in digital newsletters, reports and on the Be-Life website.

##### Monitoring the external and internal environment

Efforts increased to anchor, monitor and coordinate the program with relevant actors and projects within SALAR (e.g. the pharmaceutical and legal expert groups, the patient safety project) through SALAR’s numerous networks for municipal and county councils. Efforts were also made to create a dialogue with SALAR’s political organisation and to collaborate with the National Board of Health and Welfare on appraisal of the regional results on indicators. The growing complexity of the program and the increasing number of sub-projects contributed to higher demands on program coordination. The program management team participated in 26 national and numerous regional conferences, seminars, educational activities and network meetings. Members of the team attended international conferences and made study visits abroad.

##### Building the management team

A journalist, a web assistant and an expert on pharmaceuticals joined the project management team in February 2012.

##### Internal program support, monitoring, evaluation and feedback

In order to get support, input and feedback on their work the program management team continued to use mentoring support, with regular meetings with consultants, experts and ‘critical friends’. Annual evaluation reports by the Swedish Agency for Public Management fed back information that was discussed within the team. Feedback meetings were also held with the research group. The reference group of elderly citizens took on a more active role, participating in various activities and meetings describing their views and experiences.

##### Specific monitoring and analysis of target area ‘coordinated health and social care’

One of SALAR’s new responsibilities during this period was to develop a monitoring system for the indicators ‘avoidable inpatient care’ and ‘readmission within 30 days’ and to coordinate and provide support to the regions’ analytical work in these areas. To support the analysis of measurement results a team that focused on the monitoring system was organized along with a network of regional analysts. A web-based tool for monitoring of patients readmitted to hospital within 30 days and patients discharged from hospitals was developed (in collaboration with Blekinge Institute of Technology), tested and launched in the regions during this period.

### Strategies used to facilitate program implementation

**Figure 2:**
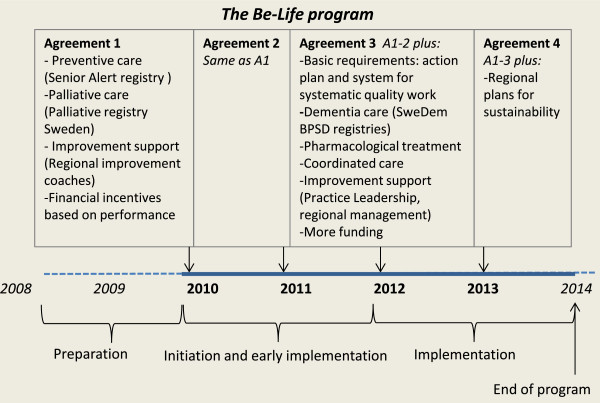
**Overview of the BeLife program.**

**Figure 3:**
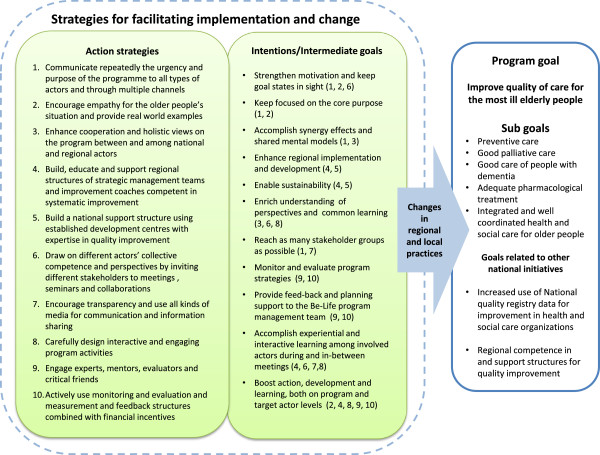
**Strategies used by the Be-Life program management to facilitate implementation, change and sustainability.**

Ten main strategies to facilitate implementation were derived (Figure 2) based on the extensive list of activities and the respondents’ descriptions of the strategic choices made during the program. The first strategy concerns the repeated communications of the urgency, purpose and progress of the program to all types of actors and through multiple channels (1). The second strategy, to encourage empathy for the most ill elderly people’s situation and provide real world examples (2), focuses on motivating and engaging people by using real-world examples of problematic and heart-warming situations. The third strategy, to enhance cooperation and holistic views on the program between and among national and regional actors (3), is exemplified by the coordination between several national initiatives and involvement of actors from these initiatives. The fourth strategy, to establish regional support structures of strategic level management teams and improvement coaches competent in systematic improvement (4) is an example of the use of regional change agents and a ‘coaching the coaches’ strategy at two hierarchical levels in the regions.The fifth strategy, to build a national support structure using established county council improvement and development centres with expertise in quality improvement (5) to provide strategic support to the regional support structures (see 4) was initiated as a part of the ‘Practice Leadership’ initiative. The aim of strategy 4 and 5 was also to enhance sustainability beyond the program on both regional and national levels. The sixth strategy, to draw on different actors’ collective competence and perspectives by inviting different stakeholders to meetings, seminars and collaborations (6), including elderly people and politicians, indicates an openness for discussion and perspective taking. The national quality registries and the analytical experts were also actively involved in many meetings and in coaching the staff that were to work with improvement and quality registries. The seventh strategy, to encourage transparency and use all kinds of media for communication and information sharing (7) is connected to strategy one, but the focus here is on being open and transparent with uncertainties, good and bad results, changes of strategies, and problems and mistakes and the attempts to act on them. The eighth strategy, to carefully design interactive and engaging program activities (8), reflects the program management team’s basic assumptions on how to foster change, by enhancing interaction, motivation and learning. The ninth strategy, to engage experts, mentors, evaluators and critical friends (9) reflects the active involvement of knowledgeable individuals and groups for various types of support to the program management team. Finally, the tenth strategy, directed mainly at the regions, concerns how to actively use monitoring, evaluation, measurement and feedback structures in combination with governmental financial incentives (10). This is exemplified by the web-portal giving access to registry data, and the provision of tools, models and reports to aid regional actors’ comprehension of measurement and results (Figure 3).

### Program outcomes

In Table 1 a selection of indicators used to measure the Be-Life program outcomes in the five target areas are presented.

**Table 1:** **Be-Life program outcomes: preventive, palliative and dementia care 2009-2013**

Senior alert registry	2009 n (%)	2010 n (%)	2011 n (%)	2012 n (%)	2013 n (%)
Municipalities listed in the registry (total N = 290)	7 (2)	196 (68)	261 (90)	280 (97)	284 (98)
County councils listed in the registry (total N = 21)	8 (38)	14 (67)	19 (90)	21 (100)	21 (100)
Municipalities with registered risk assessments for 90% of patients in special housing units for elderly people				73 (25)	129 (44)
Risk assessments - Total no of registrations	13 415	48 267 (+260)	151 787 (+214)	247 017 (+63)	256 129 (+4)
Risk assessments - No of registrations on average per month	1118	4022 (+260)	12 649 (+214)	20 585 (+63)	23 284 (+4)
**Palliative Registry Sweden**					
Municipalities listed in the registry (N = 290)	187 (64)	276 (95)	284 (98)	289 (99)	290 (100)
County councils listed in the registry (N = 21)	20 (95)	21 (100)	21 (100)	21 (100)	21 (100)
*Registered deaths (65 years and older)*					
Municipalities that reached the goal level of ≥40% coverage		221 (76)			
Municipalities ≥50%			229 (79)		
Municipalities ≥70%				174 (60)	191(66)
County councils that reached the goal of ≥40% coverage		17 (81)			
County councils - ≥50%			18 (86)		
County councils - ≥70%				15 (71)	16 (76)
**Swedish Dementia Registry (SveDem)**					
Primary care units listed in the registry (N = 2009/1095, 2010/1181, 2011/1197, 2012/1172, 2013/1172)	41 (4)	40 (3)	96 (8)	658 (56)	809 (70)
No of diagnostic work-ups performed in primary care	1026	1716 (+67)	2745 (+60)	4283 (+56)	3020 (−29)
**Registry for Behavioural and Mental Symptoms in Dementia (BPSD)**					
Municipalities listed in the registry (N = 290)		3 (1)	27 (9)	206 (71)	265 (91)
No of registrations in the BPSD registry		123	1138 (+825)	7142 (+527)	24 040 (+237)
No of registered patients		77	542 (+604)	3894 (+618)	11 613 (+198)

#### Preventive care

Before the program, the Senior Alert registry was virtually unknown among the municipalities. During the first two years the utilization of the registry increased dramatically. There was a similar development among the county councils. In 2010 and 2011, performance bonuses were related to the number of registered risk assessments. From 2012 additional bonuses were granted to municipalities where a risk assessment was performed for a minimum of 90 percent of the elderly people living in special housing units. During the first two years, the average number of registered risk assessments per month increased by over 200 percent each year.

#### Palliative care

At program start the Palliative Registry Sweden was well-known among municipalities and county councils and by the second year, almost all municipalities and all county councils had units participating in the registry. Performance bonuses were paid if the required percentage of all registered deaths in the region was reached. The target levels for performance bonuses were gradually raised each year. The proportions of municipalities and county councils reaching the target levels have fluctuated between 60–79 percent (municipalities) and 71–89 percent (county councils) over the program period. From 2012, bonuses were also granted based on whether the patient and/or their relatives received verbal information about the imminent death, a key activity in the palliative care process.

#### Dementia care

When the SveDem registry was included in the program in 2012 most patients that were given diagnostic work-ups at specialized dementia care units was being registered, but the coverage within primary care was very low. During the first year in the program, the number of participating primary care units and number of registered diagnostic work-ups increased substantially. There was an even more prominent development regarding the utilization of the registry for Behavioural and Mental Symptoms in Dementia in municipalities, where the number of registered patients increased from 77 to 11 613 between the years 2010 and 2013.

#### Pharmacological treatment

All regions reduced their use of the specified medications during 2012, but only two regions (10%) reached the target of a ten percent reduction. To receive performance bonuses in 2013, the regions had to reduce the prescription of the three types of medications for at least four months during the six month long measurement period (April-August 2013), as compared to the same period in 2012. Patient data from registries, based on place of residence (i.e. municipality), were used and aggregated to county level. All regions managed to reduce prescription of inappropriate medications and anti-inflammatory medications, and two thirds of the regions reached the goal related to medications used to treat psychoses. Variation in results on municipality level is presented in Table 2. Thus, even regions with high adherence to target levels had municipalities that did not reach them.

**Table 2:** **Be-Life program outcomes: pharmacological treatment 2012-2013**

	No and proportion of municipalities (N = 290) that reduced prescription April-August 2013							
		0 months	1 month	2 months	3 months	4 months	5 months	6 months
Inappropriate medications	No	0	2	3	13	19	42	211
	%	0	0,7	1	4	7	14	73
Anti-inflammatory medications	No	1	9	15	21	35	54	155
	%	0,3	3	5	7	12	19	53
Antipsychotic medications	No	49	29	27	38	28	56	63
	%	17	10	9	13	10	19	22

Number and proportion of municipalities that reduced the prescription of targeted medications during the measurement period April-August 2013 compared to April-August 2012.

#### Coordinated health and social care

In 2012 no region reached the target level of a ten percent reduction in the coordinated care index (calculated from the two indicators ‘avoidable inpatient care’ and ‘readmission within 30 days’). A majority of the regions (16 of 21) showed small reductions, less than 2 percent. The two regions with the largest reductions reduced their index levels by 7 and 5 percent respectively. Two other regions increased their coordinated care index by 7 and 9 percent respectively. However, large variations between the regions’ monthly results could be found. In 2013, the criteria were refined and performance bonuses paid to the regions that showed a statistically significant improved monthly value for at least four months, as compared with the same period in 2012, for the two indicators respectively. Ten regions (48%) reached the goal for the indicators ‘avoidable inpatient care’ and another ten the goal of ‘readmission within 30 days’. Three regions (14%) reached the goals for both indicators. Results on national level are presented in Table 3.

**Table 3:** **Be-Life program outcomes: coordinated health and social care 2012-2013**

National level	Year	Jan	Feb	Mar	Apr	May	Jun	Jul	Aug	Sep	Oct	Nov	Dec
Avoidable care events	2012	9 165	9 044	9 768	9 000	9 224	8 717	8 458	8 464	8 384	9 234	9 085	8 896
	2013	9 737	8 569	9 268	9 608	9 321	8 113	8 399	7 982	8 204	9 201	8 492	8 206
Total care events with diagnosis	2012	58 345	57 401	62 509	57 433	59 602	55 103	51 060	54 284	55 898	61 625	60 495	56 256
	2013	61 177	55 395	59 627	60 359	60 566	52 665	51 450	51 911	55 616	61 461	56 650	52 035
Proportion of avoid-able inpatient care events (%)	2012	15,7	15,8	15,6	15,7	15,5	15,8	16,6	15,6	15,0	15,0	15,0	15,8
	2013	15,9	15,5	15,5	15,9	15,4	15,4	16,3	15,4	14,8	15,0	15,0	15,8
Readmissions day 1–30 - care events	2012	10 194	10 033	11 058	10 300	11 001	10 298	10 177	9 955	9 640	10 992	10 830	10 366
	2013	10 612	9 920	10 524	10 769	11 046	9 959	10 078	9 518	9 759	10 507	9 698	9 851
Total care events	2012	60 979	59 483	64 619	59 779	62 064	57 208	53 042	55 999	58 019	63 635	62 940	58 729
	2013	63 210	57 498	61 664	62 523	62 705	54 820	53 226	53 880	57 727	62 219	58 385	57 818
Proportion of read-missions day 1–30 (%)	2012	16,7	16,9	17,1	17,2	17,7	18,0	19,2	17,8	16,6	17,3	17,2	17,7
	2013	17,3	17,3	17,1	17,2	17,6	18,2	18,9	17,7	16,9	16,9	16,6	17,0

## Discussion

The Be-Life program involved a massive amount of activities directed at many different groups of actors during its first three years. As reflected in our case description, the program included a mixture of intended and emergent strategies as a large part of the program was built during the process and only some aspects were systematically prepared and planned for at the time for the initiation of the program. The program was organized as a project (i.e. limited in time and organized and implemented by a team that eventually will be resolved) with the regional organizations responsible for achieving and sustaining changes and improvements. When a project organization is used to implement policies it is vital to incorporate a complete understanding of the multiple levels of actions needed and the different kinds of variables that can be expected to influence both output and outcome [36]. It is also important to recognize that the initial idea from the government, namely to combine several initiatives for a larger coherent program approach towards the most ill elderly people, provided a proper base for SALAR to choose a more holistic strategic approach aimed at multiple actors and hierarchical levels.

The program management provided visions for the future situation for elderly people that pleaded to peoples’ values and life situation and referred to the gap between the current situation and the vision in several areas. The program aims and indicators were primarily related to changes in care procedures and in the elderly person’s situation. Aims for phases in the change process were less articulated, for example how good support structures and procedures should develop over time. The interventions included conventional actions proven successful, but also new rather untested and overarching measures. The program was supported by a solid management structure with strong and charismatic leadership. Through the agreements, the Be-Life program and SALAR were provided with substantial monetary resources for coordinating and supporting the implementation and change process. These results are all in line with the ‘Planning and infrastructure’ driver in Perla et al’s model [15], which includes vision and aim, carefully developed interventions, solid management, and sufficient resources.

Perla et al’s model [15] also highlights the need to ‘*Consider individual, group, organizational, and system factors’*. The program activities and strategies were directed at many different individuals and groups, sometimes inviting several organisational functions, such as higher level managers in multiple areas, to get synergy effects. At national meetings with regional strategic managers various techniques for enhancing learning and interaction were used – in smaller or larger groupings. The regional support structures can also be seen as learning networks, social networks and includes the involvement of local champions and change agents. The many meetings, educational activities, seminars and web sites focused on the aim of building regional capability and capacity. The important role of leadership during change and development was emphasized by the launch of the ‘Practice leadership’ initiative, involving key decision makers in regional organizations. The program management team emphasized values, but organizational culture was not directly confronted, more indirectly approached when cooperation between care providers was addressed. Facilitation of the implementation required dealing with the diversity of sub-systems and their varying capabilities. This required separate meetings, visits and coaching activities in the regions – which were frequently carried out by the program management team. The many activities aimed for the many different actors and stakeholders more or less involved all eight secondary drivers in Perla et al.’s model [15]. One aspect worth mentioning was the initial lack of strategies, both on national and regional levels, on how to reach the private care providers. This might be due to the relatively recent development of opportunities to choose care provider in Sweden. Reaching private actors will need more attention and further adaption of program strategies, as the provider organisations are either small businesses or organisations with a large geographical spread of units that are not as easily fitted into a regional approach.

The pace of change in LSTs is relatively slow as it often is difficult to have a clear plan from the beginning, it demands lots of involvement from many stakeholders and the main attempt of change agents is to minimize resistance [29, 30]. The Be-Life program strategies fits well with strategies described by Kotter and Schlesinger [29, 30] for achieving change during complex circumstances: using education and communication, participation and involvement and facilitation and support, sometimes combined with negotiation and agreement.

*‘The process of change’* driver in Perla et al’s model [15] is the heart of a change program and can be approached in different ways. The Be-Life program was actively pushed by its management team who used a mix between a help strategy and a make-it-happen strategy to achieve change. Perla et al. [15] did not specify any change theory or theories in the framework. In the Be-Life program we found indications of several slightly overlapping change theories in terms of de Caluwé and Vermaak’s colour categorization of basic views on change [24, 25]. In the first phase the focus was on negotiation in order to gain support and delegation to act. This resembles the strategy of emphasizing negotiation and consideration of many stakeholders’ views and can be considered a natural first step in the initiation of change programs [26]. This so called Yellow-print strategy continued in the second phase as the program goal, its main content and actors were negotiated and decided on. It was complemented by the more dominating interactive learning strategy, the Green-print strategy, which allows for trials and experimentation. The Green-print strategy continued to be strong in the implementation phase in combination with an increased amount of rational planning and structuring, activities emphasized by the Blue-print strategy.

With regard to the third dimension of this driver, mechanisms used for spread, Perla and colleagues [15] emphasizes the lack of a clear synthesis or taxonomy of frameworks for large-scale spread and sustainability. This makes it hard to address this dimension. Perla and colleagues suggests that complex interventions that have not been extensively tested might benefit from more collaborative methods of spread. The Be-Life program’s use of several underlying views and theories on change involved both top-down strategies, such as the Blue-print, as well as mixed or bottom-up strategies involving a higher degree of interaction, such as the Green- and Yellow-print strategies. These strategies also indicate underlying assumptions about how to achieve change – by focusing on individuals’ needs, interaction and learning, systematic procedures, networking and creating a favourable situation for change.

From the start, the Be-Life program had a *‘System for performance measures and evaluation’* in place – driver four in Perla et al.’s model [15]. The national quality registries included in the program provided an infrastructure that involved the registry holders, their web sites and their expertise. Measures on regional levels were regularly provided by the registries on a web site. The measurement and feedback system were further developed during the program period and made more easily accessible for regional actors. In addition, the program’s progress was regularly followed and measured by external actors and fed back to the program management. The use of monitoring and feedback strategies that can follow the program’s development process, progress, output and outcomes are often overlooked, but necessary to enhance development [37]. Using structured ways of assessing the current quality situation in health care organisations, and involving many actors while doing so, can enhance the readiness for change in national programs [38].

Sizable amounts of money from the state were invested in the Be-Life program, as the regions’ performance was connected to financial incentives. Resources were also used to build regional support structures and to support improvement of registry output and competence in analyses. The application of financial incentives to support changes in healthcare has often targeted micro levels, such as general practitioners, physicians and patients e.g. [39, 40]. In Sweden financial incentives have been used more recently in several national change initiatives directed at regions and provider organisations, but all aspects of these reforms have not yet been assessed. A recent study on financial incentives as driving force for improvements in healthcare indicated that finances do play a role, but aspects such as attention to improvements from management, dedicated staff, and practice-based projects can be equally or even more important for successful outcomes [41]. It is too early to evaluate the impact of the financial incentives in the Be-Life program, but the structured observations made of discussions among the regional actors during meetings indicate that the incentives influenced the motivation to act and participate in the program, at least on political and higher management levels.

The analysis of outcome data shows a positive development in the use of the quality registries in the areas of preventive, palliative, and dementia care. Results for the indicators on pharmacological treatment were also positive. As could be expected, effects on the system level indicators of coordinated of care were more modest, so far. Based on these results we infer that the Be-Life program during the studied period has had an impact on care for the elderly citizens in line with its goals in all of the five target areas. Results on national level also show that change happened faster in some areas (e.g. preventive care) than in others (e.g. coordinated care). These differences can be related to many factors like pre-existing work with registries, the complexity level of the target area and the number of involved care-giver organisations. More specific investigations are needed to address the program outcomes and the long-term effects on regional level, as well as to understand the mechanisms for more or less successful regional or local implementation.

### Methodological considerations

This study is limited to the first four years of the Be-life program leaving one year yet to enhance progress towards the goals. Yet, longitudinal mixed methods studies of LSTs have a potential to expand our understanding of change strategies in complex systems. Such studies can also address the understudied aspects of sustainability and long-term impact [13]. A follow-up of the Be-Life program can shed more light on the potential of the program strategies described in this study. There is also a need for a coordinated, multilevel model or taxonomy with a certain degree of flexibility for describing LST strategies used in complex systems, in order to be able to compare cases systematically. Multiple case studies of LSTs using a comparative framework can further enhance our understanding of the nature of strategies used to facilitate the implementation and change in large scale change programs. The way of presenting the strategies in this study resemble the concept ‘theory of change’ and its use within program theory [42, 43]. In this study we describe the program management team’s views, strategies and approaches, with less focus on the program as an entity, even though the two are related. We also suggest the use of de Caluwé and Vermaak’s colour categorization of basic views on change [24, 25] as one way to describe underlying change theories in driver three of Perla et al’s model [15].

## Conclusions

This case study presents an innovative approach used for implementing a large national change program characterized by an initial flexibility and dynamism regarding content and facilitation strategies, as well as a growing complexity that required increased structuring and coordination as it developed over time. The description of activities and strategies showed that the program management team engaged a variety of stakeholders and accordingly they developed and used a broad array of strategies to facilitate the implementation of the program content. There is no blue-print on how to approach the challenging task of leading large change programs in complex contexts, but our conclusion is that more research and practical focus is needed on the multidimensionality of strategies that program management needs to consider. The strategies used in the Be-Life program depended on the program content, contextual factors, system levels, types of actors and their influences, the program’s progress over time, types of learning and change processes inferred, and the conditions for sustainability. These aspects can give some indications on some of the basic aspects of the strategic multidimensionality needed for achieving LSTs in health and social care.

## Abbreviations

Be-Life: Better life for the most ill elderly people program in Sweden
BPSD: The registry for Behavioural and Mental Symptoms in Dementia
LST: Large-system transformations
Palliative registry: A quality registry focusing on improving palliative care
Practice Leadership: “From word to action – the practice of leadership”, a sub part of the Be-Life program focusing on support to higher-level managers and key actors in the regions
SALAR: The Swedish Association of Local Authorities and Regions
Senior Alert: A quality registry focusing on the prevention of falling accidents, malnutrition, decubitus ulcers, and poor oral health
SveDem: The Swedish Dementia Registry.
